# Spatial distribution of Parkinson's disease mortality in Spain, 1989-1998, as a guide for focused aetiological research or health-care intervention

**DOI:** 10.1186/1471-2458-9-445

**Published:** 2009-12-02

**Authors:** Jesús de Pedro-Cuesta, Eduard Rodríguez-Farré, Gonzalo Lopez-Abente

**Affiliations:** 1 Department of Applied Epidemiology, National Centre for Epidemiology, and Consortium for Biomedical Research in Neurodegenerative Diseases (Centro de Investigación Biomédica en Red sobre Enfermedades Neurodegenerativas - CIBERNED), Carlos III Institute of Health. C/ Sinesio Delgado 6. 28029 Madrid. Spain; 2 Environmental Health Group, Consortium for Biomedical Research in Epidemiology & Public Health-Carlos III Institute of Health (CIBERESP-ISCIII), Barcelona, Spain; 3 Department of Pharmacology and Toxicology, Barcelona Institute of Biomedical Research (Instituto de Investigaciones Biomédicas de Barcelona -IIBB), Scientific Research Board-August Píi Sunyer Biomedical Research Institute (Consejo Superior de Investigaciones Científicas-Institut d'Investigacions Biomèdiques August Píi Sunyer:CSIC-IDIBAPS), Rosellón 161, E-08036 Barcelona, Spain; 4 Environmental and Cancer Epidemiology Unit, National Centre for Epidemiology, Carlos III Institute of Health, C/ Sinesio Delgado 6. 28029, Madrid, Spain; 5 Consortium for Biomedical Research in Epidemiology & Public Health (CIBERESP), C/ Sinesio Delgado 6. 28029, Madrid, Spain

## Abstract

**Background:**

Aetiologically, genetic and environmental factors having an uneven spatial distribution may underlie Parkinson's disease (PD). Undiagnosis of PD in selected regions might have limited access to treatment with levodopa and simultaneously, if present at death, determined PD underreporting at the death record. The purpose of this study was to describe and analyse municipal mortality due to PD in Spain in aetiological and interventional perspective.

**Methods:**

PD mortality at a municipal level was modelled using the Besag-York- Molliè autoregressive spatial model, combining demographic information with cause-of-death diagnostic data (International Classification of Diseases 9^th ^Revision (ICD-9) code 332.0). Municipal relative risks (RRs) were independently estimated for women, men and both sexes, and plotted on maps depicting smoothed RR estimates and the distribution of the posterior probability of RR>1.

**Results:**

A south-north gradient, with large geographical areas suggesting clustered towns with high mortality, was seen in Asturias, the Basque Country, Balearic Islands and, particularly, in the Lower Ebro valley around Tarragona. Similarly, there was a suggestion that lowest mortality was clustered in the south-east and south-west. We identified some isolated or clustered municipalities with high mortality that were situated near industrial plants reported to be associated with environmental xenobiotic emissions. However, the same pattern was also observed for some cities with low mortality.

**Conclusion:**

Municipal PD mortality in Spain was unevenly distributed. Patterns were roughly similar to reported provincial PD mortality and use of levodopa. While the overall pattern appears to result from spatially selective PD undiagnosis, and can not be ascribed to industrial emissions, it can not be excluded that selected "hot spots" reflect genetic factors and/or environmental exposures inducing parkinsonism. A few municipal populations, located in low-mortality-risk areas in the vicinity of polluting plants or registering high excess PD mortality, might constitute a priority for conducting direct etiological studies. Additionally, interventions aimed to reduce potential PD undiagnosis might be most appropriate in the South.

## Background

The purpose of this study was to detect spatially uneven mortality from Parkinson's disease (PD) in Spain as a tool potentially useful for design of focused etiological research and interventions aimed to reduce possible PD undiagnosis and undertreatment. The first aim requires a detailed positioning with regard to causality in PD. The second one, will consider comparisons with geographical patterns of levodopa use (LDU).

The aetiology of the most common forms of Parkinson's Disease (PD), whether sporadic or familial, is poorly understood. Genetic heterogeneity, with at least eight susceptibility loci, has been implicated in rare, monogenic, familial forms [1]. Nevertheless, low concordance in twins [2], familial-aggregation patterns [3], birth-cohort effects [4], and the results of diverse genome-wide linkage and association studies [1,5,6], albeit debated, support the contention that the large majority of sporadic PD cases result from a synergistic effect of multigenic inheritance and environmental factors. The nature of Lewy bodies, a hallmark of late-onset PD neurodegeneration, consisting of deposits of aggregated misfolded α-synuclein and other proteins through the dysfunctioning of α-synuclein and other genes, may, in part, have elucidated molecular phenomena responsible for monogenic familial and some sporadic PD forms [7,8]. Exposure to combined environmental chemicals, such as pesticides and metals, may alter α-synuclein and dopaminergic function in the substantia nigra [9-12]. In addition, a mitochondrial complex I function defect --described in brain tissue and platelets of patients affected by sporadic PD-- has been proposed as a plausible pathophysiological apoptotic mechanism shared by most PD forms [13-15]. Through activation of apoptotic molecular pathways, mitochondrial poisons, such as 1-methyl-4-phenyl-1,2,3,6-tetrahydropyridine (MPTP) and rotenone, lead to degeneration of substantia nigra pars compacta, of a type similar to that seen in PD [16,17]. Likewise, many other oxidative stress-inducing agents lead to activation of mitochondrial apoptotic pathways [10]. Recently-published animal model studies are consistent with a complex aetiology for late-onset PD, i.e., multigenic susceptibility paving the way for a neurodevelopmental basis plus environmental toxic multi-insults or ageing [18]. However, evidence from PD natural history or epidemiology which supports specific steps, is sparse.

Clinical and experimental MPTP observations in the early 1980s fuelled the search for environmental toxins potentially implicated in PD; paraquat, a herbicide chemically similar to MPTP, was perhaps the first of a series of such hypotheses relating to various manmade toxins, with pesticides being by far the most frequently investigated [9,10,12,19]. A detailed review of five cohort studies, 38 separate case-control studies and one meta-analysis on pesticide risk, supported by even more recent findings [20], concluded that there does indeed appear to be evidence of a potential role of pesticides in the development of PD, with the current body of evidence being insufficient to establish causation for any specific pesticide [21]. Conversely, an ubiquitous natural toxin, Pertussis toxin, mediated by high age at infection, or dietary exposure to marine food contaminants has been proposed as aetiological environmental factors explaining birth-cohort effects in Iceland and high PD prevalence in geographical isolates, i.e., in Iceland, Greenland and the Faroe Islands [19,22,23].

Studies able to indicate geographical regions with a potentially high incidence of PD can be crucial for planning analytical surveys or case-control studies aimed at testing specific hypotheses on industrial pollutants, such as pesticides, insecticides and heavy metals claimed to be potentially relevant for the disorder [24]. The publication of European Pollutant Emission Register-Spain (EPER-Spain) data enables the presence of geographical patterns linked to industrial pollution to be investigated [25,26]. In Europe, Spain has been the leading polluter in almost one third of all EPER pollutant substances released into the environment, and ranks among the top three leading polluters in two-thirds of all such substances. In addition use of pesticides in agriculture has been historically important in Spain. For a number of years, however, the only nation-wide source of PD diagnostic and residential data has been the cause-of-death registry. A recent disease-mapping mortality study in Spain, in which PD was included, disclosed an unexpected spatial pattern for this disorder [27].

This paper thus sought to: describe and map the municipal distribution of parkinsonism-related mortality in Spain; discuss possible determinants of patterns; and indicate the most promising study populations for the purpose of undertaking direct studies (which tend to be expensive) aimed at investigating the relationship between industrial pollutant emissions and PD or to reduce PD undiagnosis. This study did not quantitatively analyse the relationship between industrial pollutants and PD mortality.

## Methods

Spanish municipal populations, broken down by age group (18 groups) and sex, were obtained from the 1991 census and 1996 municipal roll. These years correspond to the midpoints of the two quinquennia that comprise the study period (1989-1993 and 1994-1998). The person-years for each five-year period were obtained by multiplying these populations by 5.

For our case source, we used individual death entries for the period 1989-1998, corresponding to Parkinson's disease (International Classification of Diseases 9^th ^Revision (ICD-9) code 332.0) as the underlying cause of death, broken down by town or city, nation-wide. These data were furnished by the National Statistics Institute (*Instituto Nacional de Estadística *- *INE*) for the production of a municipal cancer mortality atlas of which these results form part [27].

Standardised mortality ratios (SMRs) were calculated as the ratio of observed to expected deaths. For the calculation of expected cases, the overall Spanish mortality rates for the above two 5-year periods were multiplied by each town's person-years, by age group, sex and quinquennium.

For map-plotting purposes, smoothed municipal relative risks (RRs) were calculated, using the conditional autoregressive model proposed by Besag, York and Molliè (BYM). This model was introduced by Clayton and Kaldor [28], developed by BYM [29], and subsequently applied in the field of ecological studies [30]. These models are based on fitting Poisson spatial models with observed cases as the dependent variable, expected cases as offset, and two types of random effects terms which take the following into account: a) municipal contiguity (spatial term); and b) municipal heterogeneity. The models were fitted using Markov chain Monte Carlo simulation methods with non-informative priors [31]. Posterior distributions of relative risk were obtained using WinBugs [32]. The criterion of contiguity used was adjacency of municipal boundaries. Convergence of the simulations was verified using the Bayesian Output Analysis (BOA) R programme library [33]. Given the great number of parameters of the models, the convergence analysis was performed on a randomly selected sample of 10 towns and cities, taking 4 strata defined by municipal size. Convergence of the estimators was achieved before 100,000 iterations. For the maps shown, a "burn-in" (iterations discarded to ensure convergence) of 300,000 iterations was performed and the posterior distribution was derived with 5,000.

A Geographic Information System was used to plot municipal maps that depicted smoothed RR estimates and the distribution of the posterior probability (pp) that RR>1 (Bayesian version of p value). With regard to this indicator, we followed Richardson's criterion [34], which recommends that probabilities above 0.8 should be deemed significant.

## Results and Discussion

From 1989 to 1998, a total of 12531 PD deaths were registered in Spain, 6311 in men and 6220 in women. Summary statistics for population and PD deaths in 8077 municipalities are shown in Table 1. In 5266 towns and cities no death due to this cause was registered. Using these data it was possible to ascertain the posterior distribution of relative risk on the basis of a single spatial model that included all of Spain's 8077 towns and cities and the 46398 adjacencies existing between them.

**Table 1:** Summaries of population and PD mortality in Spain's 8077 towns: 1989-1998.

	Number	Mean	Median	Standard deviation	Minimum	Maximum	No. (%) of areas with zero counts
Population	39960592	4947.45	594	42459,34	5	2866850	0 (0)

Observed PD	12531	1.55	0	14.53	0	888	5266 (65.2)

Expected PD	13168	1.63	0.357	15.59	0	1078.41	2 (0)

PD SMRs	-	0.83	0	2.10	0	43.48	5264 (65.2)

**Figure 1:**
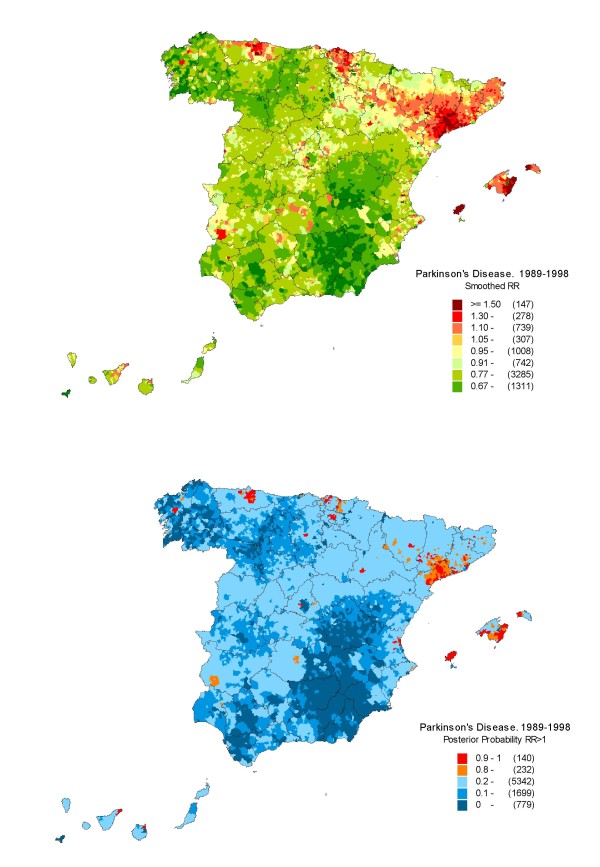
**Municipal distribution of Parkinsonism mortality in Spain: 1989--1998**. *Upper*: Distribution pattern of smoothed relative risk (RR) under the BYM model. *Lower*. Posterior probability of RR being greater than 1. Spain 1989--1998.

Figure 1 depicts the distribution of: a) the smoothed RRs for PD (both sexes): and b) the posterior probability (pp) that RR>1. This second map "filtered" the first, by flagging the areas in which excess mortality was more likely. Since sex-specific patterns were similar [27], the results for both sexes were graphically depicted here.

While the most striking spatial high-mortality pattern corresponded to the north-eastern area of the Lower Ebro valley in Catalonia, centred on Tarragona, the lowest mortality was observed in the south-east, in an area covering part of the provinces of Jaén, Granada, Almería, Albacete and Murcia, and in the south-west, in the provinces of Seville and Cadiz. All three patterns would suggest a clustering of several municipalities. Other large geographic areas to register the highest mortality were located in northern provinces, in regions such as Asturias and the Basque Country, as well as in the Balearic Islands.

Figures for towns with RRs of over 1.3 after smoothing and a posterior probability >0.80 of having an RR >1, are shown in Table 2 grouped by province. Unlike Pozuelo de Alarcon (Madrid), six out of seven towns with RRs >2, such as Cambrils, Montblanc and Tarragona in the Lower Ebro valley and Felanixt, Eivissa and Santa Eulalia in the Balearic Islands, appeared to come within "hot spots"., Montblanc, however, was the only town displaying peak RR values >2, which had at least one EPER industry at a distance of less than 2 km from the municipal centroid. Fifteen out of 40 towns with higher-than-expected values in the table had a minimum of one EPER industry at a distance of less than 2 km from the municipal centroid.

**Table 2:** Municipalities with 10 or more observed deaths due to Parkinson Disease, RRs >= 1.3 and a posterior probability >= 0.80 of having an RR greater than 1.

Province	*INE *code	Town/Municipality	Observed number of deaths	Expected number of deaths	SMR	RR
Alava	1059	Vitoria-Gasteiz*	78	56.9	1.37	1.32

						

Asturias	33024	Gijón*	143	94.4	1.51	1.49

	33035	Llanera*	11	4.4	2.51	1.68

	33037	Mieres del Camino*	33	18.8	1.75	1.54

	33044	Oviedo*	100	71.5	1.40	1.37

	33051	Pravia	11	5.6	1.97	1.44

	33060	San Martín del Rey Aurelio	19	7.9	2.41	1.82

	33066	Siero	22	16.3	1.35	1.31

	7033	Manacor	24	11.2	2.14	1.90

	7054	Santa Eulalia del Río	10	4.8	2.10	2.04

						

Barcelona	8019	Barcelona*	888	678.8	1.31	1.30

	8056	Castelldefels	23	7.8	2.94	2.26

	8096	Granollers	25	13.9	1.80	1.51

	8102	Igualada	24	12.6	1.91	1.65

	8112	Manlleu	10	5.3	1.87	1.45

	8125	Montcada I Reixac*	12	6.8	1.77	1.32

	8187	Sabadell	84	57.9	1.45	1.40

	8266	Cerdanyola del Vallès*	14	9.0	1.55	1.30

	8298	Vic	17	11.3	1.51	1.35

	8305	Vilafranca del Penedès*	16	9.9	1.63	1.47

	8307	Vilanova I la Geltrú*	22	15.1	1.45	1.44

						

A Coruña (Corunna)	15001	Abegondo	10	3.2	3.12	1.36

	15078	Santiago de Compostela	40	25.1	1.59	1.31

						

Gerona	17015	Banyoles	11	5.5	2.01	1.61

	17114	Olot	23	12.8	1.80	1.51

	17117	Palafrugell	11	6.3	1.74	1.37

						

Guipuzkoa	20055	Arrasate O Mondragon	10	6.7	1.49	1.33

						

Huesca	22048	Barbastro*	12	6.6	1.82	1.37

						

Madrid	28080	Majadahonda	14	5.2	2.69	1.651

	28115	Pozuelo de Alarcón	29	9.8	2.95	2.12

						

Murcia	30005	Alcantarilla	14	6.8	2.06	1.59

						

Tarragona	43038	Cambrils	14	4.1	3.41	2.21

	43086	Montblanc*	12	2.5	4.77	2.46

	43148	Tarragona*	48	32.1	1.50	1.48

	43161	Valls	22	8.1	2.73	2.18

						

Valencia	46085	Carlet	15	5.7	2.64	1.51

	46244	Torrent	33	14.0	2.35	1.73

						

Vizcaya	48036	Galdakao*	11	6.0	1.83	1.42

	48044	Getxo*	31	22.4	1.38	1.33

						

Zaragoza	50067	Calatayud	19	9.0	2.12	1.48

* A minimum of one EPER industry at a distance of less than 2 km from municipal centroid.

Shown in Table 3 are the figures for municipalities with 5 or more expected deaths, RRs <1.0 after smoothing and a posterior probability <= 0.10 of having an RR >1 (mortality deficit), regardless of the number of observed deaths. Lowest values, RR <0.6, were seen for 12 towns with populations ranging from 8891 in Ortigueira (Corunna) to 85884 in San Fernando (Cadiz). A considerable proportion, 32/72, of such towns, had a minimum of one EPER industry at a distance of less than 2 km from the municipal centroid.

**Table 3:** Towns with lower-than-expected PD mortality. Towns with 5 or more expected deaths from Parkinsonism and a posterior probability <= 0.10 of having an RR greater than 1

Province	*INE *code	Town/Municipality	Observed number of deaths	Expected number of deaths	SMR	RR
Albacete	2009	Almansa*	1	7.28	0.14	0.62

	2081	Villarrobledo*	1	6.42	0.16	0.55

Alicante	3011	Alfaz del Pi	0	5.71	0	0.59

	3031	Benidorm	1	10.71	0.09	0.52

	3133	Torrevieja	3	10.46	0.29	0.59

Barcelona	8015	Badalona*	42	52.60	0.80	0.84

	8101	Hospitalet de Llobregat (L')	58	71.91	0.81	0.82

	8211	Sant Feliu de Llobregat*	1	8.35	0.12	0.69

Burgos	9219	Miranda de Ebro*	4	12.36	0.32	0.66

Cadiz	11006	Arcos de La Frontera*	2	5.76	0.35	0.64

	11012	Cadiz	25	36.76	0.68	0.67

	11015	Chiclana de La Frontera	4	7.64	0.52	0.66

	11020	Jerez de La Frontera*	25	38.65	0.65	0.68

	11031	San Fernando	6	16.48	0.36	0.53

	11032	Sanlúcar de Barrameda	7	10.66	0.65	0.71

Castellón	12040	Castellón de La Plana/Castello*	26	41.37	0.63	0.69

CiudadReal	13039	Daimiel	3	6.05	0.49	0.74

	13053	Manzanares	1	6.30	0.16	0.63

	13082	Tomelloso	5	9.82	0.51	0.65

	13087	Valdepeñas	6	8.79	0.68	0.71

	14021	Cordoba*	61	80.90	0.75	0.76

Cordoba	14056	Puente Genil	5	7.93	0.63	0.74

Corunna (*A Coruña*)	15005	Arteixo*	0	5.15	0	0.60

	15019	Carballo	5	8.89	0.56	0.68

	15036	Ferrol	23	31.18	0.72	0.73

	15054	Narón*	6	9.43	0.64	0.69

	15061	Ortigueira	1	6.10	0.16	0.59

	18023	Baza	1	6.88	0.14	0.54

Granada	18089	Guadix*	5	5.50	0.91	0.72

Huelva	21072	Valverde del Camino	1	5.26	0.19	0.63

Jaén	23002	Alcalá la Real	3	8.20	0.37	0.64

	23005	Andujar*	7	10.16	0.69	0.76

	23055	Linares*	13	15.56	0.84	0.75

	23060	Martos	5	7.86	0.64	0.72

	23087	Torredonjimeno*	3	5.04	0.59	0.73

	23092	Ubeda	3	9.22	0.32	0.55

León	24089	León*	33	52.78	0.62	0.66

	24115	Ponferrada*	15	19.34	0.77	0.77

Logroño	26036	Calahorra	2	7.32	0.27	0.75

Lugo	27028	Lugo	25	32.51	0.77	0.77

	27057	Sarriá*	5	6.96	0.72	0.73

	27066	Viveiro	4	6.58	0.61	0.70

Madrid	28007	Alcorcón	10	24.91	0.40	0.64

	28065	Getafe	19	25.18	0.75	0.81

	28079	Madrid*	732	1078.4	0.68	0.68

Malaga	29067	Malaga*	120	133.03	0.90	0.89

Murcia	30003	Aguilas	4	6.99	0.57	0.65

	30015	Caravaca de La Cruz	2	8.28	0.24	0.55

	30016	Cartagena*	34	45.53	0.75	0.76

	30017	Cehegín	4	5.32	0.75	0.71

	30022	Jumilla	3	6.72	0.44	0.70

	30024	Lorca*	17	21.38	0.79	0.74

	30039	Totana	3	6.81	0.44	0.66

Navarre	31201	Pamplona/Iruña	48	63.13	0.76	0.77

Ourense	32019	Carballino (O)	0	6.01	0	0.57

Las Palmas	35026	Telde*	8	13.47	0.59	0.75

Pontevedra	36042	Ponteareas	1	5.47	0.18	0.55

	36052	Silleda	2	5.56	0.36	0.67

	36055	Tui	2	5.59	0.36	0.58

	36057	Vigo*	60	76.29	0.78	0.77

Santander	39075	Santander*	57	69.22	0.82	0.83

Seville	41004	Alcalá de Guadaira*	9	11.28	0.80	0.75

	41024	Carmona*	2	6.99	0.28	0.62

	41038	Dos Hermanas*	10	15.64	0.64	0.72

	41053	Lebrija	3	5.52	0.54	0.69

	41060	Marchena*	2	5.31	0.37	0.67

	41065	Morón de La Frontera*	3	8.06	0.37	0.63

	41091	Seville*	147	184.31	0.80	0.80

Teruel	44216	Teruel*	10	11.96	0.84	0.78

Valencia	46022	Alfafar	1	5.03	0.20	0.71

	46105	Cullera	3	7.40	0.41	0.72

	46131	Gandía	8	14.66	0.55	0.72

* A minimum of one EPER industry at a distance of less than 2 km from municipal centroid.

**Figure 2:**
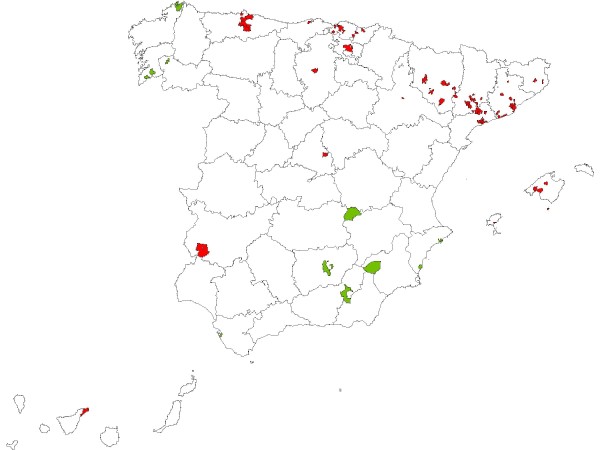
**Geographical location of selected data**, i.e., municipalities with higher-than-expected RR values, whether or not included in Table 2, with a minimum of one EPER industry at a distance of less than 2 km from municipal centroid (red), and 12 municipalities listed in Table 3 registering lowest, RR <= 0.6 values (green).

The above-mentioned 12 towns listed in Table 3, which exhibited the lowest values, RR <= 0.6, and towns with higher-than-expected RR values, whether or not included in Table 2, which had at least one EPER industry at a distance of less than 2 km from the municipal centroid, are pinpointed in the map in Figure 2. In general, low-mortality towns (shown in green) were grouped in the north-east and south-west. A considerable number of towns with high mortality and at least one EPER industry at a distance of less than 2 km from the municipal centroid (shown in red) were located in the above-mentioned "hot spots" in the north, and in the north-east along the northern sector of the Ebro basin. However, a few others, such as Jerez de los Caballeros (Badajoz) in the south-west, Tenerife (Canary Islands) and Alcalá de Henares (Madrid) corresponded to distinct, isolated areas, seen against a low-mortality background in Figure 1.

## Discussion

To our knowledge, this study is the first ever attempt to disclose differences in the spatial distribution of PD mortality in populations resident in small areas nation-wide. Results suggest that there is a considerable variation, with a south-north gradient and some potential clusters. Differences should be associated with PD or PD-like disorders, since PD is the most frequent type of parkinsonism. Nevertheless, they could be explained by other multiple single or combined factors, such as ascertainment, reporting, or specific aetiological factors, including potential exposure to environmental contaminants and persistent organic pollutants in particular.

Mortality statistics on PD are difficult to interpret because reporting bias may not only be frequent but may also change with time and place. A review [24] pertinent to our study period reported that: 1) in clinical series, the proportion of PD sufferers whose death certificates made no mention of the disease ranged from 68% in Iceland to 81% in Rochester; and 2) in those cases where PD was reported, the annual proportion recorded as the underlying cause of death in Sweden ranged from 46% to 81%, and varied by county. The shift that took place in Sweden in the early 1980s from recording PD as a contributory cause of death to recording it as the underlying cause of death, was attributed to late implementation of a World Health Organisation (WHO) recommendation: even if this had been present in Spain, where the underlying cause of death was studied, it nevertheless occurred before our study period and so could not affect these results. Changes in death-certificate coding of PD during the study period would generate an effect perceived at the level of administrative regional boundaries, something that is not seen in the maps. An internationally described PD mortality pattern, observed for instance in the USA, Sweden, the UK and Spain, corresponds to an ubiquitous increase with advanced age and a decrease at lower ages [24]. In Spain, age-period-cohort analysis revealed an increase in both sexes as a period effect since 1970, and as an inverted-U cohort effect with highest mortalities for cohorts born around 1910 and lowest mortalities in recently-born cohorts [35]. We propose that, since mapped PD mortality would mainly reflect mortality among the elderly, the geographical pattern might be principally determined by specific changes across time-spatially sensitive, i.e., non-simultaneous, improvement in medical services in terms of diagnosing late-onset PD.

Since prevalent undiagnosed PD as seen from Spanish population screening surveys ranged from 12% to 69% [36], and PD incidence in a Spanish population aged 65 years and over [37] was ten times higher than PD mortality yielded by our study, PD diagnosis reported at death probably had a low sensitivity for PD. Low death-record sensitivity for PD might be due to low PD ascertainment at death, low reporting bias or low mortality/fatality of PD patients. Since all-cause mortality in the general population is higher in the south [27], it would seem difficult to explain the south-north pattern in terms of the effect of high general mortality on PD patients.

**Figure 3:**
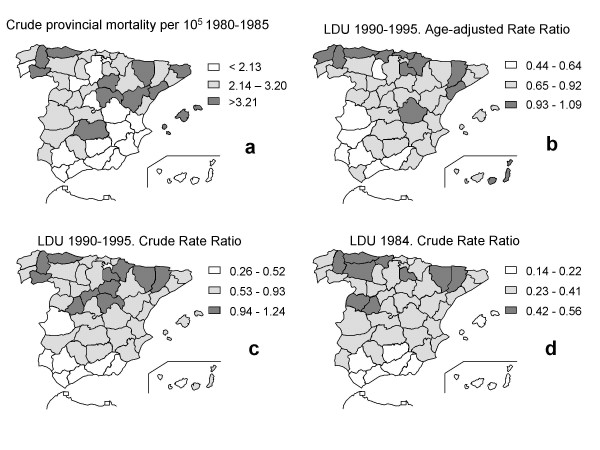
**Spatial patterns of reported provincial PD mortality (a) and use of levodopa (b, c, and d) in Spain**.

To a certain extent, the PD mortality pattern described fits that reported for LDU and provincial PD mortality [38,39] (Figure 3). In 1992, Bruguera et al. [38] had already noticed the similarity between PD mortality in the 1980-1985 period and the provincial LDU pattern in 1984 [39]. Cuadrado et al analysed age-adjusted LDU from 1990 to 1995, and reported high drug sales in the northern coastal areas and in Catalonia, and low drug sales in the south and south-west of the country [40], which fits the municipal pattern shown here rather well. In brief, it would appear that the major features of spatial distribution of municipal PD mortality in Spain correspond to geographical differences seen for LDU, shared by both sexes, and persisting across time. Since prescription of levodopa, a relatively disease-specific therapy, and reporting PD as a cause of death require PD diagnosis, it would appear that PD aetiology, PD underascertainment or both constitute potential explanations for the spatial patterns observed by us, thereby rendering resort to potential PD reporting bias in death records unnecessary.

Despite the fact that a higher incidence of PD among men has been documented in a Spanish population [37], the similar geographical mortality pattern among men and women (not shown here) would point to causes of geographical variation determined by incidence unconnected with occupation. It would be difficult for differential misdiagnosis of PD, such as overdiagnosis (i.e., low specificity), to underlie spatial variation, since PD-like forms of parkinsonism are excluded as a potential explanation due to their considerably lower prevalence [Spanish Group for Epidemiological Study of the Aged, unreported].

Door-to-door PD prevalence surveys have been conducted on four Spanish populations [41-45]. All of these displayed medium prevalence, except the town of Cantalejo in the Province of Segovia, where the high prevalence detected among the elderly segment aged over 80 years was attributed to detailed and intensive case-finding [45]. None of these populations was located in any of the mortality "hot spots" identified by us. Neither unreported PD surveys conducted in the Hondarribia district in the Basque Country [46], Arosa Island. [47] and selected subpopulations in Central Spain with medium prevalences [Spanish Groups for Epidemiological Study of the Aged, unreported], nor a survey based on diagnoses collected by the medical services in Lower Aragon, a region lying close to the north-eastern cluster [48], are of any help when it comes to interpreting our mortality findings for specific towns.

While EPER industries were located in the vicinity of many towns with high or low PD mortality, the excess mortality registered in many towns in Catalonia, the Basque Country, Huesca, Asturias and Tarragona is consistent with the traditional, well-documented industrial toxic emissions reported in these places [26,27]. The mortality cluster in the Province of Tarragona warrants special attention, since the province encompasses the easternmost stretch of the Ebro basin which historically accumulated the industrial pollution borne by the Ebro River, potentially acting via water and, essentially, through the food chain and diet. Tens of thousands of tons of waste, consisting mainly of several hexachlorocyclohexane (HCH) isomers, were dumped near factories in the Basque Country and the Province of Huesca, namely, in Barakaldo from 1947-1987, in Erandio from 1952-1982 and in Sabiñanigo, with the HCH isomers being disseminated by lixiviation and environmental conditions, and being detected in tributaries of the Ebro River [49]. For over a century, an electrochemical plant and a chemical factory located at Flix have polluted the river and the region with organochlorine compounds and mercury. This pollution has been detected in the ecosystems of the Ebro River delta area [50,51] and in human serum [52,53], with high levels of hexachlorobenzene and other organochlorine compounds being found during pregnancy and in cord blood [54-56]. Despite the fact that the chemical factories and waste areas are situated close to or not very far from the PD "hot spots" described here, our findings align better with historical exposures of the type reported in the EPER involving emissions from multiple industrial plants near the city of Tarragona, in which lead, cadmium and other organic and inorganic industrial chemicals are included [57].

However, some results for PD mortality clusters also deviate from biologically expected patterns. Firstly, low PD mortality is seen in environmentally polluted areas, such as in Huelva, or in the Gibraltar area in southern Spain where both cancer mortality and registered industrial pollution are high [26-28,58]. Secondly, the lack of excess cancer mortality seen in towns in the Province of Tarragona is difficult to reconcile with high, historically long-lasting industrial pollution and high PD mortality. Third, the lower mortality in Benidorm and the Canary Islands contrasts with the high mortality in the Balearic Islands where the possible weight of the foreign, aged, European resident population should be considered.

Attempts have occasionally been made to link parkinsonism to industrial pollutants but hypotheses (those targeting heavy metals being frequently mentioned) lack confirmation, generally as well as specifically, in the form of recent gene-environment interaction analysis [59]. Manganese, carbon disulphide, carbon monoxide after acute poisoning, cyanide, n-hexane and other chemical toxicity may include features mimicking parkinsonism [60-62]. Exposure to β-HCH has recently been reported as a risk factor for PD [12]. Were one to speculate, PD mortality clusters in northern Spain and Catalonia might be consistent with Gorell et al's hypothesis, suggesting that there is a large array of compounds that may alter the nigrostriatal dopaminergic pathway and induce neural oxidative stress, leading to the development of PD [10] in which the reported effects of ubiquitous organohalogen xenobiotics --mainly organochlorines-- and polychlorinated biphenyls (PCBs) might be implicated [11,63-66]. In contrast, a few of the above-mentioned towns with high mortality deserve particular attention because they were located in areas of low industrial activity and low PD mortality, displaying a history of isolated and selective environmental industrial pollution at a distance of less than 2 km from their municipal centroids, such as Jerez de los Caballeros (Badajoz) in the south-east and Alcalá de Henares (Madrid) in central Spain [25,26]. While familial parkinsonism similar to that found in a small community in the Province of Soria [67] might account for isolated spots of high PD mortality, the towns with high mortality listed in Table 2 have larger populations. However, there are other reasons for caution, particularly when interpreting such high mortality values in industrial areas. Because EPER facilities are registered insofar as the emissions levels exceed certain threshold levels, absence of EPER facilities in the database does not necessarily mean that industrial emissions are low. In several countries or regions within the EU, there might be a concentration of small to medium size industries, none attaining the threshold for any single pollutant, and where cumulative total emissions are high. Hence caution should be given not to equate absence of EPER facility in a given city and "low industrial activity". In summary, high mortality and absence of EPER registered facility does not exclude a concealed local positive association. To add, SMR values are affected by population size in the town targeted.

Undiagnosed PD present until death, determining PD underreporting at death certificate and low PD mortality, should be suspected as a health-relevant explanation of the lowest PD mortality observed in two broad areas, one covering tracts of several provinces in the south-east, such as Granada, Jaén and Almería, and another in the south-west corresponding to Seville and Cadiz. Such areas displayed some of the lowest LDU rates from 1990 to 1995 [40], a period encompassed by our own study period, and [39] the low PD mortality might also indicate historically-persistent low access to PD diagnosis and treatment in such regions, perhaps determined by lack of neurologists at district hospital clinics in Andalucia. Several large towns situated in the above-mentioned and other north-eastern provinces pinpointed in Figure 3 or in neighbouring areas, might be proposed for primary care intervention using reported procedures designed to uncover the potential limitations of medical services in diagnosing and treating PD [68,69].

Internal migration during the 1960s and 1970s to Madrid, Catalonia/Barcelona and the Basque Country from poor rural areas mainly located South and West [70], fitting well with regions with low LDU and PD mortality, would have induced patterns opposite to those observed. Neuroepidemiological PD research in such areas is still sparse.

## Conclusion

This study provides a summarised report of spatial patterns of municipal PD mortality roughly fitting those of reported provincial mortality and levodopa use, with municipal "hot spots", whether or not clustered, indicating populations of possible interest for direct studies, purpose-designed to test specific PD aetiological hypotheses or improving access to PD diagnosis and treatment.

## Competing interests

The authors declare that they have no competing interests.

## Authors' contributions

JPC and GLA conceived the project. GLA performed the calculations. JPC drafted first manuscript. ERF evaluated toxicological aspects. GLA and ERF contributed to final version of manuscript. All authors read and approved the final manuscript.

## Pre-publication history

The pre-publication history for this paper can be accessed here:

http://www.biomedcentral.com/1471-2458/9/445/prepub

## References

[B1] Martínez M Brice A Vaughan JR Zimprich A Breteler BMM Meco G Genome-wide scan linkage analysis for Parkinson's disease: the European genetic study of Parkinson's disease J Med Genet 2004 41 900 907 10.1136/jmg.2004.022632 15591275 PMC1735631

[B2] Wirdefeldt K Gatz M Bakaysa SL Fiske A Petzinger GM Widner H Complete ascertainment of Parkinson disease in the Swedish Twin Registry Neurobiol Aging 2008 29 1765 1773 10.1016/j.neurobiolaging.2007.04.009 17532098 PMC2662365

[B3] Sveinbjörnsdottir S Hicks A Jónsson T Pétursson H Guðmundsson G Frigge M Familial aggregation of Parkinson's disease in Iceland N Engl J Med 2000 343 1765 1770 10.1056/NEJM200012143432404 11114315

[B4] de Pedro-Cuesta J Petersen IJ Stawiarz L High levodopa use in periodically time-clustered, Icelandic birth cohorts. A vestige of parkinsonism etiology? Acta Neurol Scand 1995 91 79 88 7785429 10.1111/j.1600-0404.1995.tb00412.x

[B5] Clarimon J Scholz S Fung HC Hardy J Eerola J Hellström O Conflicting results regarding the semaphorin gene (SEMA5A) and the risk for Parkinson disease Am J Hum Genet 2006 78 1082 1084 10.1086/504727 16685660 PMC1474095

[B6] Maraganore DM de AM Lesnick TG Strain KJ Farrer MJ Rocca WA High-resolution whole-genome association study of Parkinson disease Am J Hum Genet 2005 77 685 693 10.1086/496902 16252231 PMC1271381

[B7] Spillantini MG Schmidt ML Lee VM Trojanowski JQ Jakes R Goedert M Alpha-synuclein in Lewy bodies Nature 1997 388 839 840 10.1038/42166 9278044

[B8] Gasser T Mendelian forms of Parkinson's disease Biochim Biophys Acta 2009 1792 7 587 96 19168133 10.1016/j.bbadis.2008.12.007

[B9] Corrigan FM Wienburg CL Shore RF Daniel SE Mann D Organochlorine insecticides in substantia nigra in Parkinson's disease J Toxicol Environ Health 2000 59 229 234 10.1080/009841000156907 10706031

[B10] Gorell JM Peterson EL Rybicki BA Johnsson CC Multiple risk factor in Parkinson's disease J Neurol Sci 2004 217 169 174 10.1016/j.jns.2003.09.014 14706220

[B11] Mariussen E Fonnum F Neurochemical targets and behavioral effects of organohalogen compounds: An update Crit Rev Toxicol 2006 36 253 289 10.1080/10408440500534164 16686424

[B12] Petersen MS Halling J Bech S Wermuth L Weihe P Nuielsen F Impact of dietary exposure to food contaminants on the risk of Parkinson's disease Neurotoxicology 2008 29 584 590 10.1016/j.neuro.2008.03.001 18455239

[B13] Parker WD Jr Boyson SJ Parks JK Abnormalities of the electron transport chain in idiopathic Parkinson's disease Ann Neurol 1989 26 719 723 10.1002/ana.410260606 2557792

[B14] Schapira AH Cooper JM Dexter D Clark JB Jenner P Marsden CD Anatomic and disease specificity of NADH CoQ1 reductase (complex I) deficiency in Parkinson's disease J Neurochem 1990 54 823 827 10.1111/j.1471-4159.1990.tb02325.x 2121905

[B15] Keeney PM Xie J Capaldi RA Bennett JP Jr Parkinson's disease brain mitochondrial complex I has oxidatively damaged subunits and is functionally impaired and misassembled J Neurosci 2006 26 5256 5264 10.1523/JNEUROSCI.0984-06.2006 16687518 PMC6674236

[B16] Vila M Przedborski S Targeting programmed cell death in neurodegenerative diseases Nat Rev Neurosci 2003 4 365 375 10.1038/nrn1100 12728264

[B17] Perier C Tieu K Guegan C Caspersen C Jackson-Lewis V Carelli V Complex I deficiency primes Bax-dependent neuronal apoptosis through mitochondrial oxidative damage Proc Natl Acad Sci USA 2005 102 19126 19131 10.1073/pnas.0508215102 16365298 PMC1323177

[B18] Barlow BK Cory-Slechta DC Richfield EK Thiruchelvam M The gestational environment and Parkinson's Disease: evidence for neurodevelopmental origins of a neurodegenerative disorder Reproductive Toxicology 2007 23 457 470 10.1016/j.reprotox.2007.01.007 17350799

[B19] Langston JW Ballard PA Tetrud JW Irwin I Chronic parkinsonism in humans due to a product of meperidine analog synthesis Science 1983 219 979 980 10.1126/science.6823561 6823561

[B20] Dick FD Seaton A Haites N Semple SE Dick S Prescott GJ Environmental risk factors for Parkinson's disease and parkinsonism: the Geoparkinson study Occup Environ Med 2007 64 666 672 10.1136/oem.2006.027003 17332139 PMC2078401

[B21] MRC Institute for Environment and Health Pesticides and Parkinson's Disease -- A Critical Review (Web Report W21) 2009 http://www.le.ac.uk/ieh/Ref Type: Electronic Citation

[B22] de Pedro-Cuesta J Gudmundsson G Abraira V Gudmundsson G Love AT Tulinius H Whooping cough and Parkinson's disease Int J Epidemiol 1996 25 1301 1311 10.1093/ije/25.6.1301 9027539 PMC7108570

[B23] Wermuth L Joensen P Bünger N Jeune B High prevalence of Parkinson's disease in the Faroe Islands Neurology 1997 49 426 432 9270572 10.1212/wnl.49.2.426

[B24] Pedro-Cuesta J Studies on the prevalence of Paralysis Agitans by tracer methodology Acta Neurol Scand 1987 75 101 106 10.1111/j.1600-0404.1987.tb07902.x 3303809

[B25] EPER 2006 http://eper.ec.europa.eu/eper/Ref Type: Electronic Citation

[B26] García-Pérez J Boldo E Ramis R Pollán M Pérez-Gómez B Aragonés N Description of industrial pollution in Spain BMC Public Health 2007 7 40 10.1186/1471-2458-7-40 17376231 PMC1847682

[B27] López-Abente G Ramis R Pollán M Aragonés N Pérez-Gómez B Gómez-Barroso D Atlas municipal de mortalidad por cáncer en España, 1989-1998 2006 Madrid: Instituto de Salud Carlos III

[B28] Clayton D Kaldor J Empirical Bayes estimates of age-standardized relative risks for use in disease mapping Biometrics 1987 43 671 681 10.2307/2532003 3663823

[B29] Besag J York J Molliè A Bayesian image restoration, with applications in spatial statistics Annals of the Institute of Statistics and Mathematics 1991 43 1 59 10.1007/BF00116466

[B30] Clayton DG Bernardinelli L Montomoli C Spatial correlation in ecological analysis Int J Epidemiol 1993 22 1193 1202 10.1093/ije/22.6.1193 8144305

[B31] Gilks WR Richardson S Spiegelhalter DJ Markov Chain Monte Carlo in Practice 1996 London: Chapman Hall

[B32] Spiegelhalter D Thomas D Best N Gilks W Bayesian inference using Gibbs sampling. Version 0.50 1996 Cambridge: MRC: Biostatistics Unit

[B33] Smith BJ Bayesian Output Analysis Program (BOA), Version 0.99.1 for S-PLUS and R 2001 http://www.public-health.uiowa.edu/BOA Ref Type: Electronic Citation

[B34] Richardson S Thomson A Best N Elliott P Interpreting posterior relative risk estimates in disease-mapping studies Environ Health Perspect 2004 112 1016 1025 15198922 10.1289/ehp.6740 PMC1247195

[B35] López-Abente G Pollán M Aragonés N Pérez B Llácer A Pérez J Tendencias de la Mortalidad en España, 1952-1996. Efecto de la edad, de la cohorte de nacimiento y del periodo de muerte 2002 Madrid, Spain: Instituto de Salud Carlos III

[B36] Del Barrio JL de Pedro-Cuesta J Boix R Acosta J Bergareche A Bermejo F Dementia, Stroke and Parkinson's Disease in Spanish Populations: A Review of Door-to-Door Prevalence Surveys Neuroepidemiology 2005 24 179 188 10.1159/000085138 15832058

[B37] Benito-León J Bermejo-Pareja F Morales-Gonzalez JM Incidence of Parkinson's disease and parkinsonism in three elderly populations of central Spain Neurology 2004 62 734 741 15007123 10.1212/01.wnl.0000113727.73153.68

[B38] Burguera JA Catalá J Taberner P Muñoz R Mortalidad por enfermedad de Parkinson en España (1980-1985). Distribución por edades, sexo y áreas geográficas Neurología 1992 7 89 93 1571189

[B39] Limón C García IA Ortega A Utilización de levodopa en España en el periodo 1982-1984 Información Terapéutica de la Seguridad Social 1985 9 202 210

[B40] Cuadrado JI de Pedro-Cuesta J Abraira V Stawiarz L Ingesta A Almazán J Epidemiological assessment of levodopa use in Spain, 1990-1995. Persistent, low consumption in the South Pharmacoepidemiol Drug Saf 1999 8 433 445 10.1002/(SICI)1099-1557(199910/11)8:6<433::AID-PDS451>3.0.CO;2-8 15073905

[B41] de Rijk MC Tzourio C Breteler MM Dartigues JF Amaducci L Lopez-Pousa S Prevalence of parkinsonism and Parkinson's disease in Europe: the EUROPARKINSON Collaborative Study. European Community Concerted Action on the Epidemiology of Parkinson's disease J Neurol Neurosurg Psychiatry 1997 62 10 15 10.1136/jnnp.62.1.10 9010393 PMC486688

[B42] Lopez S Prevalencia de la enfermedad de Parkinson Neurología 1993 8 328

[B43] Manubens JM Martínez-Lage JM Lacruz F Prevalencia de la enfermedad de Parkinson y otro tipos de parkinsonismo en el anciano Neurología 1993 8 399

[B44] Benito-León J Bermejo-Pareja F Rodríguez J Molina JA Gabriel R Prevalence of PD and other types of Parkinsonism in three elderly populations of Central Spain Mov Disord 2003 18 267 274 10.1002/mds.10362 12621629

[B45] Clavería LE Duarte J Sevillano MD Pérez-Sempere A Cabezas C Rodríguez F Prevalence of Parkinson's disease in Cantalejo, Spain: A door-to-door survey Mov Disord 2002 17 242 249 10.1002/mds.10087 11921108

[B46] Bergareche A De la PE López de MA Sarasqueta C De AA Poza JJ Prevalence of Parkinson's disease and other types of Parkinsonism. A door-to-door survey in Bidasoa, Spain J Neurol 2004 251 340 345 10.1007/s00415-004-0333-3 15015016

[B47] Seijo-Martínez M Castro del RM Paz EJ Sobrido Gómez MJ Rodríguez Á Jr Suárez PR Parkinsonismo y enfermedad de Parkinson en la Isla de Arosa (Pontevedra): prevalencia en la población anciana Neurologia 2005 20 517

[B48] Errea JM Ara JR de Pedro-Cuesta J Prevalence of Parkinson's Disease in Lower Aragon, Spain Mov Dis 1999 14 596 604 10.1002/1531-8257(199907)14:4<596::AID-MDS1008>3.0.CO;2-U 10435496

[B49] Moreno-Zumalde J Bilbao: Declive industrial, regeneración urbana y reactivación económica 2005 Bilbao: Instituto Vasco de Administración Pública (Oñati)

[B50] Llorente GA Ferra A Ruiz X Albaigés J Accumulation and distribution of hydrocarbons, polychlorobyphenyls and DDT in tissues of three species of Anatidae from the Ebro Delta (Spain) Arch Environ Contam Toxicol 1987 16 563 572 10.1007/BF01055812 3115196

[B51] Porte C Albaigés J Bioaccumulation patterns of hydrocarbons and polychlorinated biphenyls in bivalves, crustaceans and fishes Arch Environ Contam Toxicol 1994 26 273 281 10.1007/BF00203552 8161229

[B52] Sala M Sunyer J Otero R Santiago-Silva M Grimalt J Organochlorine in the serum of inhabitants living near an electrochemical factory Occup Environ Med 1999 56 152 158 10.1136/oem.56.3.152 10448322 PMC1757711

[B53] Sunyer J Herrero C Ozalla D Sala M Ribas-Fitó N Grimalt J Serum organochlorines and urinary porphyrin pattern in a population highly exposed to hexachlorobenzene Environ Health 2002 1 1 8 10.1186/1476-069X-1-1 12495451 PMC131010

[B54] Sala M Ribas-Fitó N Cardo E de Muga ME Marco E Mazón C Levels of hexachlorobenzene and other organochlorine compounds in cord blood: exposure across placenta Chemosphere 2001 43 895 901 10.1016/S0045-6535(00)00450-1 11372882

[B55] Ribas-Fitó N Torrent M Carrizo D Júlvez J Grimalt JO Sunyer J Exposure to hexachlorobenzene during pregnancy and children's social behavior at 4 years of age Environ Health Perspect 2007 115 447 450 17431497 10.1289/ehp.9314 PMC1849941

[B56] Carrizo D Ribas-Fitó N Torrent M Sunyer J In utero post-natal accumulation of organochlorine compounds in children under different environmental conditions J Environ Monit 2007 9 523 529 10.1039/b700247e 17554423

[B57] García-Pérez J Boldo E Ramis R Pérez-Gómez B Pollán M Aragonés N Cancer mortality and industrial pollution in Spain. Abstract Book ISEE/ISEA International Conference on Environmental Epidemiology and Exposure 204

[B58] Monge-Corella S García-Perez J Aragonés N Pollán M Perez-Gómez B López-Abente G Lung cancer mortality in towns near paper, pulp and board industries in Spain: a point source pollution study BMC Public Health 2008 8 288 10.1186/1471-2458-8-288 18702814 PMC2527328

[B59] Dick FD De PG Ahmadi A Osborne A Scott NW Prescott GLJ Gene-environment interactions in parkinsonism and Parkinson's disease: the Geoparkinson study Occup Environ Med 2007 64 673 680 10.1136/oem.2006.032078 17449559 PMC2078383

[B60] Adler CH Differential diagnosis of Parkinson's disease Med Clin North Am 1999 83 349 367 10.1016/S0025-7125(05)70108-5 10093582

[B61] Tanner CM Occupational and environmental causes of parkinsonism Occup Med 1992 7 503 513 1496432

[B62] Riley DE Jankovic J, Tolosa E Secondary parkinsonism Parkinson's Disease and Movement Disorders 1998 London: Williams and Wilkins 317 339

[B63] Mariussen E Fonnum F The effect of polychlorinated biphenyls on the high affinity uptake of the neurotransmitters dopamine, serotonine, glutamate and GABA into rat brain synaptosomes and vesicles Toxicology 2001 159 11 21 10.1016/S0300-483X(00)00374-7 11250051

[B64] Seegal RF PCBs and dopamine function-Neurological effects of polychlorinated biphenyls: Does occupational exposure alter dopamine-mediated function? Parkinson's disease: The life cycle of the dopamine neuron Ann NY Acad Sci 2003 991 322 325

[B65] Seegal RF Brosch KO Okoniewski RJ Coplanar PCB congeners increase uterine weight and frontal cortex dopamine in the developing rat: implications for developmental neurotoxicity Toxicol Sci 2005 86 125 131 10.1093/toxsci/kfi174 15843507

[B66] Caudle WM Richardson JR Delea KC Guillot TS Wang M Pennell KD Polychlorinated biphenyl-induced reduction of dopamine transporter expression as a precursor to Parkinson's disease-associated dopamine toxicity Toxicol Sci 2006 92 490 499 10.1093/toxsci/kfl018 16702228

[B67] Mauri A Asensio M Jiménez A Huerta JR Redondo MJ del VV Familial Parkinson's Disease Neurología 1990 5 45 47 2361035

[B68] Mutch WJ Smith WC Scott RF A screening and alerting questionnaire for parkinsonism Neuroepidemiology 1991 10 150 156 10.1159/000110261 1922649

[B69] Sevillano-García MD Cuadrado-Gamarra JI de Pedro-Cuesta J Enfermedad de Parkinson en España: evidencias de infradiagnóstico y puntos de partida para su reducción Rev Neurol 1999 29 881 883 10696668

[B70] Migracion y estructura regional 1968 Instituto Nacional de Estadística. Madrid 55 66

